# *Fodinisporobacter ferrooxydans* gen. nov., sp. nov.—A Spore-Forming Ferrous-Oxidizing Bacterium Isolated from a Polymetallic Mine

**DOI:** 10.3390/microorganisms12050853

**Published:** 2024-04-25

**Authors:** Zhen Jiang, Xiutong Li, Zonglin Liang, Zebao Tan, Nan Zhou, Ying Liu, Zhenghua Liu, Huaqun Yin, Kun Luo, Supawadee Ingsriswang, Shuangjiang Liu, Chengying Jiang

**Affiliations:** 1 State Key Laboratory of Microbial Resources, Institute of Microbiology, Chinese Academy of Sciences, Beijing 100101, China; 2 University of Chinese Academy of Sciences, Beijing 100049, China; 3 College of Plant Protection, Hunan Agricultural University, Changsha 410128, China; 4 Key Laboratory of Biometallurgy of Ministry of Education, School of Minerals Processing and Bioengineering, Central South University, Changsha 410083, China; 5 Thailand Bioresource Research Center (TBRC), National Center for Genetic Engineering and Biotechnology (BIOTEC), National Science and Technology Development Agency (NSTDA), Pathum Thani 12120, Thailand; 6 State Key Laboratory of Microbial Biotechnology, Shandong University, Qingdao 266237, China

**Keywords:** *Alicyclobacillaceae*, *Fodinisporobacter ferrooxydans*, ferrous iron oxidation

## Abstract

A novel acidophilic, aerobic bacterium strain, MYW30-H2^T^, was isolated from a heap of polymetallic mine. Cells of strain MYW30-H2^T^ were Gram-stain-positive, endospore-forming, motile, and rod-shaped. Strain MYW30-H2^T^ grew at a temperature range of 30–45 °C (optimum 40 °C) and a pH range of 3.5–6.0 (optimum 4.0) in the presence of 0–0.5% (*w*/*v*) NaCl. Strain MYW30-H2^T^ could grow heterotrophically on yeast extract and glucose, and grow mixotrophically using ferrous iron as an electron donor with yeast extract. Menaquinone-7 (MK-7) was the sole respiratory quinone of the strain. Iso-C_15:0_ and anteiso-C_15:0_ were the major cellular fatty acids. The 16S rRNA gene sequence analysis showed that MYW30-H2^T^ was phylogenetically affiliated with the family *Alicyclobacillaceae*, and the sequence similarity with other *Alicyclobacillaceae* genera species was below 91.51%. The average amino acid identity value of the strain with its phylogenetically related species was 52.3–62.1%, which fell into the genus boundary range. The DNA G+C content of the strain was 44.2%. Based on physiological and phylogenetic analyses, strain MYW30-H2^T^ represents a novel species of a new genus of the family *Alicyclobacillaceae*, for which the name *Fodinisporobacter ferrooxydans* gen. nov., sp. nov. is proposed. The type strain is MYW30-H2^T^ (=CGMCC 1.17422^T^ = KCTC 43278^T^).

## 1. Introduction

The family *Alicyclobacillaceae*, within the class *Bacilli*, was originally described by da Costa M.S., et al. [1], and later emended by Klenk et al. [2]. Currently, the family comprises seven genera, including *Alicyclobacillus* [3], *Tumebacillus* [4], *Kyrpidia* [2], *Effusibacillus* [5], *Collibacillus* [6], *Sulfoacidibacillus*, and *Ferroacidibacillus* [7]. Members of this family inhabit a variety of environments, such as permafrost [4], soil [8], copper mines, acidic water and sediment [7,9], as well as beverages [10], and potentially play important roles in the improvement of metallurgical efficiency, the biogeochemical cycling of iron elements in acidic environments, and even the fermentation process of beverages. Cells of *Alicyclobacillaceae* are straight rods of variable length, forming terminal or subterminal ovoid endospores. The majority of the species are Gram-stain-positive, non-pigmented, mesophilic, slightly thermophilic or thermophilic, and acidophilic. Most species are aerobic and chemoorganotrophic, utilizing organic compounds as their sole carbon and energy sources. However, a few strains can reduce nitrate to nitrite, and some can even reduce Fe (III) [7,11,12]. Some species can grow mixotrophically using Fe^2+^, S^0^, and sulfide minerals as electron donors in the presence of yeast extract as a sole organic compound [7,13]. Acid is produced from several carbohydrates such as D-glucose, L-arabinose, D-xylose, D-mannose, D-Mannitol, and D-maltose by some strains. The predominant respiratory quinone of this family is MK-7. Many species possess *ω*-cyclohexane or ω-cycloheptane, yet some do not. All species have branched-chain iso- and anteiso-fatty acids and straight-chain fatty acids. The type genus *Alicyclobacillus* was first described by Wisotzkey et al. [3] and emended by a few other researchers [14], and was found in fruit juices, soils, and water of geothermal and mineral areas. To date, there are 29 validly published species under the International Code of Nomenclature of Prokaryotes (ICNP) in this genus, with species showing diverse physiological and metabolic patterns: heterotrophic or facultatively autotrophic, aerobic, or facultatively anaerobic. The genus *Tumebacillus* was identified in 2008 by Steven et al. [4], including 10 species with validated names isolated from various habitats, such as arctic permafrost [4], soil samples [15], cassava wastewater [16], decomposing algal scum [17], river water [18], and the gut of a vulture [19]. The genus *Kyrpidia* was reclassified from thermophilic, hydrogen-oxidizing *Bacillus tusciae*, and until now there were only two species in the genus; both of them were facultative autotrophs [2,20]. The genus *Effusibacillus* was established in 2014 with facultatively anaerobic or strictly aerobic chemoorganotrophic bacteria; the type species *E. lacus* could reduce nitrate to nitrite [5]. The genus *Collibacillus* was recently isolated from an athletic field in Japan, and the type species *C*. *campus* TP075^T^ is a moderately thermophilic, rod-shaped, aerobic bacterium that forms terminal endospores [6]. Ten strains of extremely acidophilic, facultatively anaerobic heterotrophic bacteria, which comprised two genera and three species, were proposed by D. Barrie Johnson in 2023 [7]. *Sulfoacidibacillus* species are mesophilic or moderately thermophilic small rods that catalyze both dissimilatory iron oxidation and iron reduction, as well as the oxidation of zero-valent sulfur and tetrathionate. *Ferroacidibacillus* species are mesophilic small rods that catalyze both dissimilatory iron oxidation and iron reduction. 

In recent years, the demand for various precious metal resources in human production and life has been increasing, and efficient and economic metallurgical technology breakthrough cannot be realized without the in situ culture of indigenous populations. In this study, we report the strain MYW30-H2^T^, isolated from a heap of polymetallic mine, and propose to establish a new genus, *Fodinisporobacter*, in the family *Alicyclobacillaceae*, based on genotypic, chemotaxonomic, and phenotypic characteristics. This study will enrich the strain bank of *Alicyclobacillaceae* and provide theoretical guidance and strain resources for improving the efficiency of the metallurgical process of polymetallic minerals.

## 2. Materials and Methods

### 2.1. Isolation and Culture Conditions

The ore samples were collected from a polymetallic mine in Guangdong province, China (N 24°33′18″, E 113°43′21″), and were incubated at room temperature in sterile water for three months. For isolation, 1 mL of the culture was used. Pure cultures were obtained using dilution plate techniques on modified B2M plates (pH 3.0) at 30 °C. The B2M medium [21] was modified by reducing yeast extract to 0.04% (*w*/*v*), supplemented with 0.04% (*w*/*v*) glucose. The purity of the colonies was confirmed by 16S rRNA gene sequencing analysis and phenotypic homogeneity using a phase-contrast microscope (Axiostar plus, Zeiss, Jena, Germany). Unless otherwise stated, cells were grown on modified B2M solid (pH 3.0) or liquid (pH 3.5) medium at 30 °C.

### 2.2. Morphological and Physiological Analysis

Cell morphology and flagella were observed using a transmission electron microscope (TEM, JEM-1400, JEOL, Akishima, Japan) with cells grown on modified B2M plates for 5–7 days. Motility was observed via optical microscopy. The presence of spores and endospores was checked using a phase-contrast microscope (Axio Imager A2, Zeiss, Jena, Germany). Gram reaction was carried out as described in [22]. The temperature range for growth was tested in the B2M liquid medium from 15 to 60 °C with a 5 °C interval. The pH range for growth was examined by adjusting the pH of the modified B2M liquid medium to 1.0–9.0 with 0.5 intervals by 3 mol/L H_2_SO_4_ or 1 mol/L NaOH. NaCl tolerance was determined at 0–5.0% (*w*/*v*) NaCl concentration in the modified B2M liquid medium with a 0.5 concentration interval. Cell growth was estimated by measuring turbidity at a wavelength of 600 nm using the microplate reader (VICTOR Nivo, PerkinElmer, Waltham, MA, USA).

Oxidase activity was determined using the OX agent in API 20NE kits, and catalase activity was tested using a 3% H_2_O_2_ reagent. Other enzyme activities were determined using the API ZYM kits. Acid production was tested using API 50CH by replacing 50 CHB/E medium with BSS (pH 4.0) [21] mixed with 0.04% (*w*/*v*) yeast extract and 0.1% (*w*/*v*) gelrite (Sigma-Aldrich, St. Louis, MO, USA); 10 mg/L bromocresol green was used as the indicator for acid production. Acid production was assessed by the color change from blue to yellow. Carbon source assimilation and other biochemical tests were performed using API 20NE kits according to the manufacturers’ instructions.

Cultures at the exponential stage were inoculated in the modified B2M liquid medium with 13.9 g/L or 2.78 g/L FeSO_4_∙7H_2_O (50 or 10 mmol/L Fe(II)), precipitated sulfur (5 g/L), 10 mmol/L Na_2_SO_3_, 10 mmol/L Na_2_S_2_O_3_, and 5 mmol/L K_2_S_4_O_6_ as potential electron donors. The concentrations of Fe(II), Fe(III), and SO_4_^2−^, and changes in pH were measured after one week up to one month to evaluate the oxidization capacities of iron and sulfur oxyanions (thiosulfate, polythionates) or elementary sulfur at oxic conditions. Fe(III) (10 mmol/L FeCl_3_∙6H_2_O), precipitated sulfur (5 g/L), and SO_4_^2−^ (10 mmol/L Na_2_SO_4_) were separately supplemented as electron acceptors in liquid medium with 0.8 g/L yeast extract as electron donors, and the color change of lead acetate test paper and the concentrations of Fe(II) or SO_4_^2−^ at anoxic conditions were examined to judge the reduction ability of the strains. The production of H_2_S was able to blacken lead acetate and the product was assayed by the lead acetate test paper (Art. No. RZK01472). The pH value was measured by a pH meter (Mettler Toledo, Greifensee, Switzerland). The concentration of Fe(II)/Fe(III) was detected using a 1,10-phenanthroline spectrophotometry assay [23] and the concentration of SO_4_^2−^ was determined using the barium sulfate turbidimetric method [24] with a portable colorimeter (DR890, HACH, Ames, IA, USA) following the instructions of the instrument.

### 2.3. Phylogenetic Analysis

The 16S rRNA gene was amplified by PCR using primers 27F and 1492R [25]. After purification, the PCR product was sequenced as described by Haseltine et al. [26]. The full-length 16S rRNA gene sequence was compared to available sequences from the EZBioCloud [27] and NCBI (www.ncbi.nlm.nih.gov accessed on 1 March 2024) data libraries. The maximum-likelihood tree based on 16S rRNA (rrs) gene sequences of the type strains within *Alicyclobacillaceae* from the GenBank database (https://www.ncbi.nlm.nih.gov/genbank/ accessed on 1 March 2024) was constructed in MEGA 7 [28] with a partial deletion of gaps (95% cut-off). Multiple sequence alignments were performed with MUSCLE [29]. Model testing was based on the lowest corrected Akaike information criterion (AICc). The General Time Reversible model was used with a gamma distribution (5 discrete gamma categories) with invariant sites (G+I) [30,31]. In each case, bootstrap values were calculated based on 300 replications.

### 2.4. Genome Sequencing and Analysis

The genomic DNA of strain MYW30-H2^T^ was extracted using the Wizard Genomic DNA Purification Kit (Promega, Madison, WI, USA) according to the manufacturer’s instructions. The genome was sequenced by the PacBio Sequel platform at Guangdong MAGiGEN Biotechnology company (www.magigen.com accessed on 1 March 2024), and the quality-filtered reads were then assembled with Unicycler [32] into a circular chromosomal contig. The genome annotation was performed using the NCBI Prokaryotic Genome Annotation Pipeline (PGAP) [33]. Genome sequences of the type strains within *Alicyclobacillaceae* were downloaded from the NCBI database (https://www.ncbi.nlm.nih.gov/genome/ accessed on 1 March 2024). The genome-based phylogenetic tree was constructed with 92 concatenated single-copy bacterial core genes among strain MYW30-H2^T^ and other related type strains in the family *Alicyclobacillaceae* using the UBCG pipeline [34]. Metabolic pathways were analyzed using the Kyoto Encyclopedia of Genes and Genomes (KEGG) [35]. Whole-genome average nucleotide identity (ANI) values were calculated with the EZbiocloud online tools ANI calculator (https://www.ezbiocloud.net/tools/ani accessed on 1 March 2024) [36], and the average amino acid identity (AAI) values were calculated using EzAAI [37]. Digital DNA–DNA hybridization (dDDH) values were calculated by the Genome-to-Genome Distance Calculator (GGDC) 3.0 online service [38].

### 2.5. Chemotaxonomic Characterization

For the analysis of cellular fatty acid, polar lipids, and isoprenoid quinones, cells were obtained from 7 days of culture in a modified B2M medium. The fatty acids were methylated and analyzed using gas chromatography (HP 6890 Series GC System; Hewlett Packard, Palo Alto, CA, USA) [39]. Total lipids were extracted and separated by two-dimensional TLC plates (20 cm × 20 cm silica gel; Merck, Rahway, NJ, USA) [40]. Chromatography was performed by using chloroform/methanol/water (65:24:4, *v*/*v*/*v*) in the first dimension followed by chloroform/methanol/acetic acid/water (80:12:15:4, *v*/*v*/*v*/*v*) in the second dimension. The total polar lipids, aminolipids, glycolipids, and phospholipids were identified by spraying 10% ethanolic molybdophosphoric acid (Sigma), ninhydrin (Sigma), α-naphthol, and molybdenum blue regents onto the plates, respectively. Isoprenoid quinones were extracted from freeze-dried cells with chloroform/methanol (2:1, *v*/*v*) and purified by TLC [41,42]. The purified quinones were identified by HPLC equipped with a ZOBAX ODS C18 column (4.6 × 150 mm; Agilent, Santa Clara, CA, USA).

## 3. Results and Discussion

### 3.1. Morphological and Physiological Analysis

**Figure 1:**
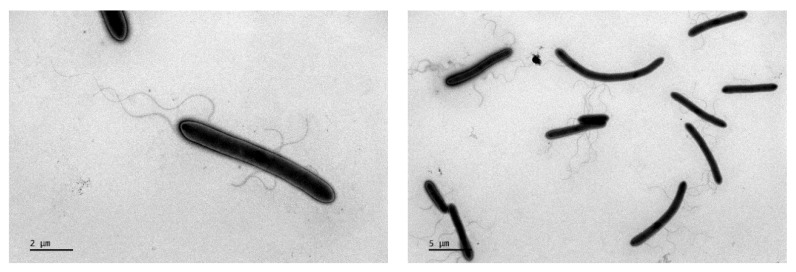
Scanning electron microscope photographs of strain MYW30-H2^T^ after growing on B2M for 7 days at 30 °C.

**Figure 2:**
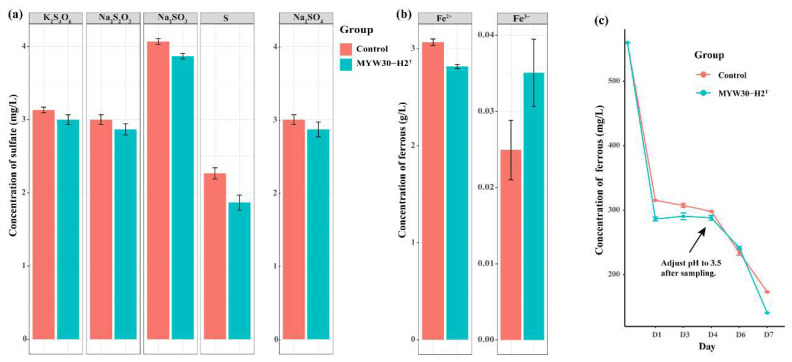
(**a**) The ability of S^0^/S_2_O_3_^2−^/S_4_O_6_^2−^/SO_3_^2−^ oxidation or SO_4_^2−^ reduction in strain MYW30-H2^T^ compared to abiotic culture detected by the accumulation or consumption of sulfate. (**b**) The ability of Fe(II) oxidation or Fe(III) reduction in strain MYW30-H2^T^ compared to abiotic culture detected by the consumption or accumulation of ferrous. (**c**) Process of iron oxidation of strain MYW30-H2^T^ compared to abiotic culture.

Cells were rod-shaped with variable lengths (0.8–1.0 μm wide and 3.8–13.7 μm long). Cells were aerobic and Gram-stain-positive, and endospores were subterminal with swollen ellipsoids. Moreover, 2–6 peritrichous flagella were observed (Figure 1). The colonies of the cell were white, opaque, rough with radial irregular edges on B2M plates, and recessed into the medium. Growth was observed at 30–45 °C (optimal 40 °C). Most species in *Alicyclobacillaceae*, except the members of *Tumebacillus*, are acidophiles, and the pH range of MYW30-H2^T^ for growth was 3.5–6.0 (optimal 4.0). Cells could grow at NaCl concentrations of 0–0.5%. The oxidase and catalase activities of strain MYW30-H2^T^ were negative. The acid phosphatase, naphthol-AS-BI-phosphohydrolase, and *α*-glucosidase activities were positive. Strain MYW30-H2^T^ was able to utilize L-Sorbose, Dulcitol, Inositol, Methyl-*α*-D-mannopyranoside, N-Acetylglucosamine, Amygdalin, Esculin, Inulin, starch, Glycogen, D-Tagatose, D-Fucose, L-Fucose, L-Arabitol, Gluconate, and 2-ketogluconate to produce acid. Assimilations of D-Glucose, L-Arabinose, D-Mannose, and D-Mannitol were observed. Nitrite production through nitrate reduction and glucose fermentation were observed. Detailed information on the biochemical characteristics of MYW30-H2^T^ is shown in Table S1. No S^0^/S_2_O_3_^2−^/S_4_O_6_^2−^/SO_3_^2−^ oxidation or S^0^/SO_4_^2−^ reduction was observed (Figure 2a). Red precipitate was observed in the ferrous sulfate culture of strain MYW30-H2^T^ and more ferrous consumption was observed in the culture of MYW30-H2^T^ compared to the abiotic culture. Subsequently, the process of iron oxidation was monitored for a week, and weak ferrous iron oxidation at a decreasing rate of 30 mg/L per day was discovered, while no Fe(III) reduction was found (Figure 2b,c). All type strains in *Alicyclobacillaceae* are Gram-stain-positive and spore-forming strains, including MYW30-H2^T^. The ability to oxidize or reduce iron or sulfur varied in *Alicyclobacillaceae*. Strain MYW30-H2^T^ and *Effusibacillus pohliae* MP4^T^ can oxidize ferrous iron. *Ferroacidibacillus* is not only an iron oxidizer in oxic conditions, but also an iron reducer in anoxic conditions. *Sulfoacidibacillus* and some of the members of *Alicyclobacillus*, like *A. tolerans* DSM 16297^T^ and *A. disulfidooxidans* DSM 12064^T^, are able to oxidize Fe(II), sulfur, and sulfide minerals like pyrite. A detailed comparison of the cell morphology, growth conditions, and substrates between strain MYW30-H2^T^ and all type strains of each genus within *Alicyclobacillaceae* is shown in Table 1.

### 3.2. Phylogenetic Analysis

**Figure 3:**
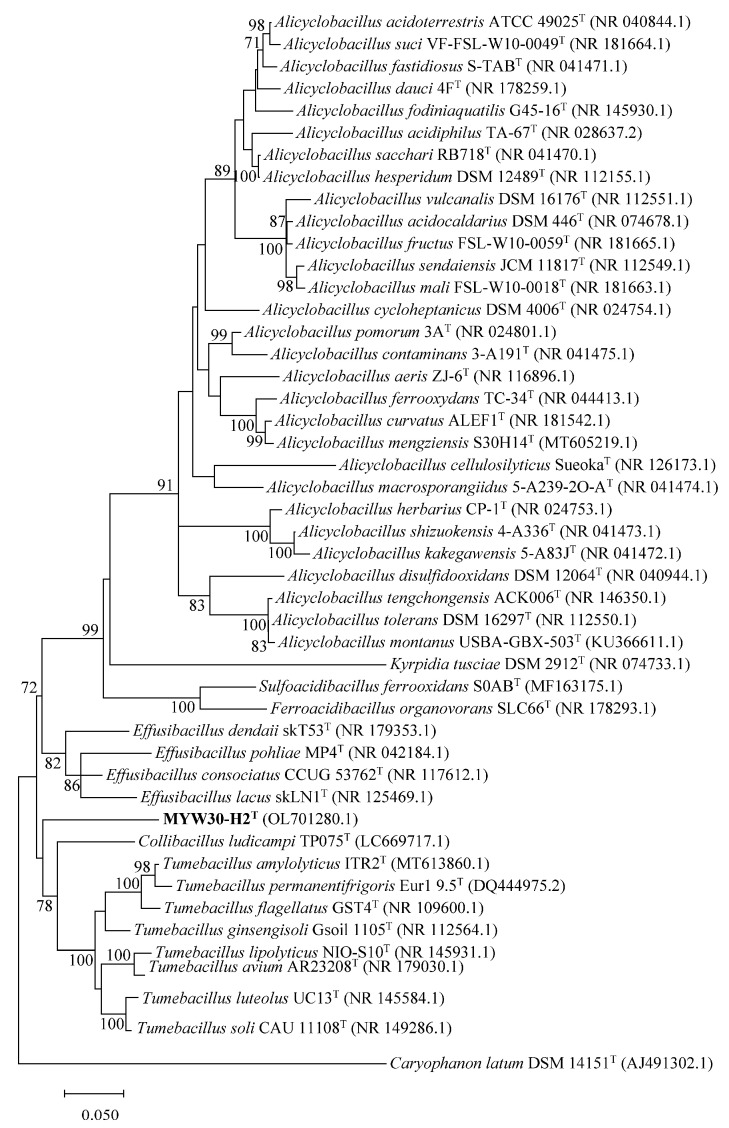
Phylogenetic tree constructed using the ML method based on 16S rRNA gene sequences of strain MYW30-H2^T^ (in bold for strain used in the present study) and other related type strains of each species in the family *Alicyclobacillaceae*. Numbers at branch nodes represent confidence levels (values ≥ 70% were shown) based on 300 replicates bootstrap samplings. GenBank accession numbers are given in parentheses. Bar, 0.050, represents the number of substitutions per site.

The 16S rRNA gene sequence length of strain MYW30-H2^T^ was 1532 bp, and it was deposited in the GenBank database under the accession number OL701280. The 16S rRNA gene-based sequence analysis showed that the strain was distinct from other genera of the family *Alicyclobacillaceae* with similarities in the range of 85.82–91.88%, and was most closely related to a member of the genus *Tumebacillus*, with the highest similarity of 91.88% to *T. avium* AR23208^T^, followed by a member of the genus *Effusibacillus*, with the highest similarity of 91.40% to *E. dendaii* skT53^T^. The same information can be obtained in the ML tree; strain MYW30-H2^T^ formed a separate branch within the family *Alicyclobacillaceae*, which indicates that the strain MYW30-H2^T^ represents a novel genus of the family *Alicyclobacillaceae* (Figure 3). The 16S rRNA gene sequence accession of MYW30-H2^T^ are listed in Appendix A.

### 3.3. Genome Characteristics

**Figure 4:**
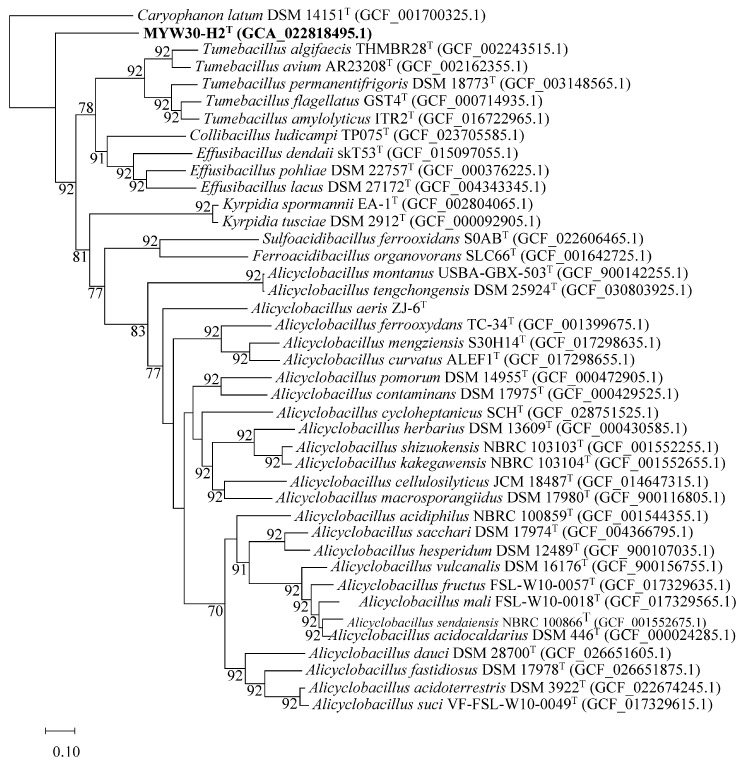
Genome-based phylogenetic tree reconstructed with 92 concatenated single-copy bacterial core genes among strain MYW30-H2^T^ (in bold for strain used in the present study) and other type strains within the family *Alicyclobacillaceae* using UBCG pipeline. Genome sequence accession numbers are given in parentheses. Numbers at branch nodes represent confidence levels; values ≥ 50% are shown. Bar, 0.10, represents the number of substitutions per site.

The genome sequence of strain MYW30-H2^T^ contained one contig and was obtained with an average coverage of 328.8×. The genome size of the strain was 4.86 Mb. The G+C content was 44.2%, which was slightly lower than the range of 44.5–66.7% for the family *Alicyclobacillaceae*. The complete genome sequence was deposited in the GenBank database under accession number CP089291. Gene prediction and annotation results showed that the genome contains 4620 genes, including 114 tRNA genes, 12 5S rRNA genes, 12 16S rRNA genes and 23S rRNA. Detailed sequencing information is shown in Table S2. The twelve 16S rRNA genes (1544 bp) in the genome showed a similarity range of 99.48–100%, with 13 base differences at most. The PCR products of the 16S rRNA genes were the same as two of the 16S rRNA genes from the genome. Pathway analyses indicated that most genes were related to metabolism, including carbohydrate, amino acid, and energy metabolism, which supported their heterotrophic growth. A phosphate acetyltransferase-acetate kinase pathway (M00579), reductive citrate cycle without citryl-CoA (M00620), and crassulacean acid metabolism (CAM) in the dark (M00168) were found in the genome, which might play a role in carbon fixation. An SOX complex (*sox*) (M00595) for thiosulfate oxidation and assimilatory sulfate reduction genes (M00176) existed in the genome, associated with the sulfur metabolism ability of the strain. The genome-based phylogenomic tree (Figure 4) also showed that strain MYW30-H2^T^ formed an independent branch in the family *Alicyclobacillaceae* and was closely related to the members of genera *Tumebacillus* and *Effusibacillus*. The ANI and dDDH values (formula 2) between MYW30-H2^T^ and other related members of the family *Alicyclobacillaceae* were 64.54–68.24% and 25.4–40.3%, respectively, indicating that strain MYW30-H2^T^ represents a novel species. Since the ANI value was not appropriate for genus delimitation [48], AAI calculations were performed and the AAI values ranged from 52.3 to 62.1% among strain MYW30-H2^T^ and other type species of family *Alicyclobacillaceae*. These values were lower than the genus boundary threshold for AAI (74%) [49], indicating that MYW30-H2^T^ represents a novel genus. The ANI, AAI, and dDDH results combined with the phylogenetic position shown by the phylogenomics tree indicated that the strain MYW30-H2^T^ belonged to the family *Alicyclobacillaceae*, but can be distinctly distinguished from the other genera of this family. A comparison of the genome features between strain MYW30-H2^T^ and all type strains of each genus within *Alicyclobacillaceae* is shown in Table 1. The genome sequence accession of MYW30-H2^T^ are listed in Appendix A.

### 3.4. Chemotaxonomic Characterization

The predominant cellular fatty acids (≥10%) for strain MYW30-H2^T^ were iso-C_15:0_ (39.1%) and anteiso-C_15:0_ (35.1%), which is similar to some extent to other genera in *Alicyclobacillaceae*. MYW30-H2^T^ has iso-C_15:0_, like the type strains of *Tumebacillus*, *Kyrpidia*, *Effusibacillus* and *Collibacillus*, and anteiso-C_15:0_, like the type strains of *Sulfoacidibacillus* and *Ferroacidibacillus*; however, it is quite different to *Alicyclobacillus acidocaldarius* 104-1A^T^ due to the complicated fatty acid profile of the genus *Alicyclobacillus.* Detailed information on cellular fatty acids components is shown in Table 2. The polar lipids profile contained a DPG, a PG, a PE, a GL, two PLs, and three unidentified polar lipids, which is similar to other genera in *Alicyclobacillaceae. Kyrpidia tusciae* T2^T^ has the same polar lipids profile as MYW30-H2^T^, while *A. acidocaldarius* 104-1A^T^ has extra phosphatidylserine. Diphosphatidylglycerol, glycolipid, and phospholipid were absent instead of phosphatidylmethylethanolamine in the type strain of *Tumebacillus* compared to MYW30-H2^T^. Phosphatidylethanolamine was absent in *Sulfoacidibacillus ferrooxidans* S0AB^T^ and *Ferroacidibacillus organovorans* SLC66^T^, but aminolipid was found in SLC66^T^. MK-7 is the only isoprenoid quinone that is uniform throughout the *Alicyclobacillaceae* family. A comparison of the chemotaxonomy compounds between strain MYW30-H2^T^ and all type strains of each genus within *Alicyclobacillaceae* is shown in Table 1. 

## 4. Conclusions

Based on phylogenetic and genomic characteristics such as phylogenetic position, a separate branch within the family *Alicyclobacillaceae* has been formed with a 16S rRNA gene sequence similarity lower than 95%, ANI value lower than 95%, dDDH value lower than 70%, and AAI value lower than 74%. Strain MYW30-H2^T^ can be distinctly differentiated from other members of the family *Alicyclobacillaceae*. On the other hand, physiological and chemotaxonomic features show that strain MYW30-H2^T^ has similar characteristics to other strains within this family, including acid production using various carbon resources, ferrous oxidation, pH, and temperature range for growth. The respiratory quinone component is MK-7, which is the same as other type strains in the family *Alicyclobacillaceae*. In conclusion, we propose that strain MYW30-H2^T^ represents a novel species of a novel genus belonging to the family *Alicyclobacillaceae*, for which the name *Fodinisporobacter ferrooxydans* gen. nov. sp. nov. is proposed.

### 4.1. Description of Fodinisporobacter gen. nov

*Fodinisporobacter* (Fo.di.ni.spo.ro.bac’ter. L. fem. n. *fodina*, mine; Gr. fem. n. *spora*, a seed and, in biology, a spore; N.L. masc. n. *bacter*, a rod; N.L. masc. n. *Fodinisporobacter*, a sporulated rod from a mine). Cells are rods, motile, mesophilic, and Gram-stain-positive. Endospores are formed. Grows heterotrophically or mixotrophically with ferrous iron and yeast extract under aerobic conditions. The respiratory quinone is MK-7. The predominant cellular fatty acids are branched-chain fatty acids iso-C_15:0_ and anteiso-C_15:0_. Phylogenetically, the genus *Fodinisporobacter* belongs to the family *Alicyclobacillaceae*. The type species is *Fodinisporobacter ferrooxydans*.

### 4.2. Description of Fodinisporobacter ferrooxydans sp. nov

*Fodinisporobacter ferrooxydans* (fer.ro.o’xy.dans. L. neut. n. *ferrum*, iron; Gr. masc. adj. *oxys*, sour or acid, used to refer to oxygen in combinations; N.L. inf. v. *oxydare*, to turn sour, to make acid, to oxidize; N.L. part. adj. *ferrooxydans*, ferrous iron-oxidizing).

Cells of strain MYW30-H2^T^ are aerobic, rod-shaped with the dimensions 0.8–1.0 × 3.8–13.7 μm, Gram-stain-positive, motile using 2–6 peritrichous flagella, have subterminal swollen ellipsoids, and are endospore-forming. Strain MYW30-H2^T^ can form white, opaque, rough colonies with radial irregular edges on B2M plates, and can recess into the medium. The temperature for growth is 30–45 °C (optimum, 40 °C). The pH range for growth is 3.5–6.0 (optimum, 4.0). Cells can grow in 0–0.5% (*w*/*v*) NaCl. Oxidase and catalase activities are negative, but acid phosphatase, naphthol-AS-BI-phosphohydrolase, *α*-glucosidase, and *β*-galactosidase are positive. Cells can utilize D-Glucose, L-Arabinose, D-Mannose, D-Mannitol, and yeast extract as carbon sources. Glucose fermentation and esculin hydrolysis are observed. Acid is produced from L-Sorbose, Dulcitol, Inositol, Methyl-*α*-D-mannopyranoside, N-Acetylglucosamine, Amygdalin, Esculin, Inulin, starch, Glycogen, D-Tagatose, D-Fucose, L-Fucose, L-Arabitol, Gluconate, and 2-ketogluconate. The strain can oxidize ferrous iron and reduce nitrate to nitrite. The predominant cellular fatty acids are iso-C_15:0_ and anteiso-C_15:0_. The polar lipids profile includes a DPG, a PG, a PE, a GL, two PLs, and three Ls. MK-7 is the only isoprenoid quinone. The DNA G+C content is 44.2%.

The type strain MYW30-H2^T^ (=KCTC 43278^T^ = CGMCC 1.17422^T^) was isolated from a polymetallic mine. The GenBank/EMBL/DDBJ accession numbers for the 16S rRNA gene and genome sequences of the type strain are OL701280 and CP089291, respectively.

## Figures and Tables

**Table 1:** Differential characteristics of strain MYW30-H2^T^ and all type strains of each genus within *Alicyclobacillaceae*. +, positive; –, negative; ND, not determined. Summed feature contains C_18:1_ω7c and/or C_18:1_ω6c. PL, unidentified phospholipid; AL, unidentified aminolipid; DPG, diphosphatidylglycerol; PG, phosphatidylglycerol; PE, phosphatidylethanolamine; GL, unidentified glycolipid; PME, phosphatidylmethylethanolamine; PS, phosphatidylserine; dDDH, digital DNA–DNA hybridization; ANI, average nucleotide identity; AAI, average amino acid identity; *, data from the present study.

	MYW30-H2^T^ *	*Alicyclobacillus acidocaldarius* 104-1A^T^ [3,21,43,44,45]	*Tumebacillus permanentifrigoris* Eur1 9.5^T^ [4,46]	*Kyrpidia tusciae* T2^T^ [2,20]	*Effusibacillus pohliae* MP4^T^ [5,47]	*Collibacillus ludicampi* TP075^T^ [6]	*Sulfoacidibacillus ferrooxidans* S0AB^T^ [7]	*Ferroacidibacillus organovorans* SLC66^T^ [7]
**Cell morphology**								
Shape	rods	rods	rods	rods	rods	rods	rods	rods
Size (μm)	0.8–1.0 × 3.8–13.7	0.7–0.8 × 2–3	0.5 × 3–3.5	0.8 × 4–5	1.5–2.5 × 0.4–0.6	0.8–1.6 × 2.4–2.7	0.5 × 2–3	0.4 × 1.5–1.8
Flagella	+	+	–	+	ND	ND	ND	ND
Oxidase	–	–	–	Weak	–	–	ND	ND
Catalase	–	+	–	Weak	–	–	ND	ND
Spore-forming	+	+	+	+	+	+	+	+
Motility	+	+	–	–	ND	–	+	+
Gram staining	+	+	+	+	+	+	+	+
**Growth conditions**								
Optimum pH	4.0	4.0	5.5–8.9	4.2–4.8	5.0	4.0–5.0	1.7	2.9
Optimum temperature	40 °C	60 °C	25–30 °C	55 °C	55 °C	47–50 °C	33 °C	30 °C
NaCl tolerance(*w*/*v*)	0.5%	4%	0.5%	1%	ND	1%	ND	ND
**Growth substrates**								
Ferrous iron	+	–	–	–	+	ND	+	+
Sulfur	–	–	–	–	–	ND	+	–
Pyrite	–	–	–	–	–	ND	+	–
Ferric iron	–	–	–	–	–	ND	+	+
**Chemotaxonomy compounds**								
Fatty acids (%)	iso-C_15:0_ anteiso-C_15:0_	Summed feature 8 * C_20:0_	iso-C_15:0_	iso-C_15:0_ iso-C_17:0_	iso-C_15:0_ iso-C_16:0_ iso-C_17:0_	iso-C_15:0_ anteiso-C_15:0_ iso-C_16:0_	anteiso-C_15:0_ anteiso-C_17:0_	anteiso-C_15:0_ anteiso-C_17:0_
Respiratory quinones	MK-7	MK-7	MK-7	MK-7	MK-7	MK-7	MK-7	MK-7
Polar lipids	DPG PG PE GL PL	DPG PG PE GL PL PS	PE PG PME	DPG PG PL PE GL	ND	ND	GL DPG PG	AL GL DPG PG PL
**Genome features (compared to S30A2^T^**)								
16S rRNA gene identities *	100%	88.52%	88.7%	87.24%	89.46%	90.64%	88.78%	87.95
Mol% G+C	44.2	60.3	53.1	59.11	55.1	46.5	46	52
dDDH *	100%	28.1%	29.3%	25.4%	26.3%	33.7%	32.3%	26.9%
ANI *	100%	64.54%	65.82%	65.88%	67.53%	68.12%	65.96%	66.24
AAI *	100%	57.09%	60.14%	58.69%	62.10%	62.39%	57.61%	58.16%

**Table 2:** Cellular fatty acid composition (% of total, in bold for main fatty acids greater than 10%) of strain MYW30-H2^T^.

Fatty Acids	MYW30-H2^T^
anteiso-C_13:0_	1.1
iso-C_14:0_	4.3
C_14:0_	1.3
**iso-C_15:0_**	**39.1**
**anteiso-C_15:0_**	**35.1**
iso-C_16:0_	2.7
C_16:0_	3.4
anteiso-C_17:0_	1.9
iso-C_17:0_	4.1

## Data Availability

Data are contained within the article and Supplementary Materials.

## Supplementary Materials

The following supporting information can be downloaded at: https://www.mdpi.com/article/10.3390/microorganisms12050853/s1, Table S1: Results of acid production (API 50CH), assimilation of carbon sources (API 20NE), enzymatic activities (API ZYM) and other biochemical characteristics (API 20NE) of strain MYW30-H2^T^. Table S2: General genomic characteristics of strain MYW30-H2^T^.

## Abbreviations

AAI, average amino acid identity; ANI, average nucleotide identity; dDDH, digital DNA-DNA hybridization; DPG, diphosphatidylglycerol; GL, unknown glycolipid; L, unidentified polar lipids; PG, phosphatidylglycerol; PE, phosphatidylethanolamine; PL, unknown phospholipids.

## Appendix A

The 16S rRNA gene sequence of strain MYW30-H2^T^ was deposited at GenBank/EMBL/DDBJ under the accession number OL701280. The genomic data of strain MYW30-H2^T^ were deposited at GenBank under the accession number CP089291 and at eLMSG (https://www.biosino.org/elmsg/index accessed on 1 March 2024) under the accession number LMSG_G000001138.1.
