# Supplementary material for: Fodinisporobacter ferrooxydans gen. nov., sp. nov.—A Spore-Forming Ferrous-Oxidizing Bacterium Isolated from a Polymetallic Mine
Source: Microorganisms. 2024 Apr 25;12(5):853. doi: 10.3390/microorganisms12050853 (PMC11123200; doi:10.3390/microorganisms12050853)
Supplement: Supplementary file 1 [file microorganisms-12-00853-s001.zip › microorganisms-2923949-supplementary.pdf]

Supplementary

Fodinisporobacter ferrooxydans gen. nov., sp. nov., a Spore-forming Ferrous-oxidizing Bacterium Isolated from a Polymetallic Mine

Zhen Jiang[1,2†], Xiutong Li[1,2†], Zonglin Liang[1,2], Zebao Tan[3], Nan Zhou[1], Ying Liu[1], Zhenghua Liu[4], Huaqun Yin[4], Kun Luo[3], Supawadee Ingsriswang[5], Shuangjiang Liu[1,2,6*], Chengying Jiang[1,2*]

[1] State Key Laboratory of Microbial Resources, Institute of Microbiology, Chinese Academy of Sciences, Beijing 100101, China

[2] University of Chinese Academy of Sciences, Beijing 100049, China

[3] College of Plant Protection, Hunan Agricultural University, Changsha 410128, Hunan, China

[4] Key Laboratory of Biometallurgy of Ministry of Education, School of Minerals Processing and Bioengineering, Central South University, Changsha 410083, China

[5] Thailand Bioresource Research Center (TBRC), National Center for Genetic Engineering and Biotechnology (BIOTEC), National Science and Technology Development Agency (NSTDA), Pathum Thani 12120, Thailand

[6] State Key Laboratory of Microbial Biotechnology, Shandong University, Qingdao 266237, China

[*]Corresponding author: Chengying Jiang, jiangcy@im.ac.cn; Shuangjiang Liu, liusj@im.ac.cn

[†]These authors contributed equally to this study.

Table S1. Results of acid production (API 50CH), assimilation of carbon sources (API 20NE), enzymatic activities (API ZYM) and other biochemical characteristics (API 20NE) of strain MYW30-H2^T. +, positive; −, negative; W, weak positive.

**Acid Production from (API 50CH):**		**Assimilation of (API 20NE):**	
Glycerol	−	D-Glucose	+
Erythritol	−	L-Arabinose	+
D-Arabinose	−	D-Mannose	+
L-Arabinose	−	D-Mannitol	+
D-Ribose	−	N-acetyl-glucosamine	−
D-Xylose	−	D-Maltose	−
L-Xylose	−	Gluconate	−
D-Adonitol	−	Capric acid	−
Methyl-*β*-D-xylopyranoside	−	Adipic acid	−
D-Galactose	−	Malic acid	−
D-Glucose	−	Trisodium citrate	−
D-Fructose	−	Phenylacetic acid	−
D-Mannose	−	**Enzymatic activities of (API ZYM):**	
L-Sorbose	+	Alkaline phosphatase	−
L-Rhamnose	−	Esterase (C 4)	−
Dulcitol	+	Esterase lipase (C 8)	−
Inositol	+	Lipase (C 14)	−
D-Mannitol	−	Leucine arylamidase	−
D-Sorbitol	−	Valine arylamidase	−
Methyl-*α*-D-mannopyranoside	+	Cystine arylaminase	−
Methyl-*α*-D-glucopyranoside	−	Trypsin	−
N-acetyl-glucosamine	+	*α*-Chymotrypsin	−
Amygdalin	+	Acid phosphatase	+
Arbutin	−	Naphthol-AS-BI-phosphohydrolase	+
Esculin	+	*α*-galactosidase	−
Salicine	−	*β*-galactosidase	−
D-Cellobiose	−	*β*-glucuronidase	−
D-Maltose	−	*α*-glucosidase	+
D-Lactose	−	*β*-glucosidase	−
D-Melibiose	−	N-acetyl-glucosaminidase	W
D-Sucrose	−	*α*-mannosidase	−
D-Trehalose	−	*β*-fucosidase	−

Inulin	+	**Other biochemical characteristics (API 20NE):**	
D-Melezitose	−	Nitrate reduction	+
D-Raffinose	−	indole production	−
Starch	+	Glucose fermentation	+
Glycogen	+	Arginine dihydrolase	−
Xylitol	−	Urea hydrolysis (Urease)	W
Gentiobiose	−	Esculin hydrolysis	+
D-Turanose	−	Gelatin hydrolysis (*β*-glucosidase)	−
D-Lyxose	−	*β*-galactosidase	+
D-Tagatose	+		
D-Fucose	+		
L-Fucose	+		
D-Arabitol	−		
L-Arabitol	+		
Gluconate	+		
2-ketogluconate	+		
5-ketogluconate	−		

Table S2. General genomic characteristics of strain MYW30-H2^T.

**Characteristics**	**MYW30-H2^T**
**INSDC number**	CP089291
**Sequencing depth**	328.8×
**Number of replicons**	1
**Genome size**	4856687 bp
**G+C content**	44.2%
**Total genes**	4620
**tRNA genes**	114
**5S rRNA genes**	12
**16S rRNA genes**	12
**23S rRNA genes**	12
**Other RNA genes**	4
